# Signal intensity informed multi‐coil encoding operator for physics‐guided deep learning reconstruction of highly accelerated myocardial perfusion CMR

**DOI:** 10.1002/mrm.29453

**Published:** 2022-09-21

**Authors:** Omer Burak Demirel, Burhaneddin Yaman, Chetan Shenoy, Steen Moeller, Sebastian Weingärtner, Mehmet Akçakaya

**Affiliations:** ^1^ Department of Electrical and Computer Engineering University of Minnesota Minneapolis Minnesota USA; ^2^ Center for Magnetic Resonance Research University of Minnesota Minneapolis Minnesota USA; ^3^ Department of Medicine (Cardiology) University of Minnesota Minneapolis Minnesota USA; ^4^ Department of Imaging Physics Delft University of Technology Delft Netherlands

**Keywords:** accelerated imaging, cardiac MRI, coil maps, deep learning, image reconstruction, myocardial perfusion

## Abstract

**Purpose:**

To develop a physics‐guided deep learning (PG‐DL) reconstruction strategy based on a signal intensity informed multi‐coil (SIIM) encoding operator for highly‐accelerated simultaneous multislice (SMS) myocardial perfusion cardiac MRI (CMR).

**Methods:**

First‐pass perfusion CMR acquires highly‐accelerated images with dynamically varying signal intensity/SNR following the administration of a gadolinium‐based contrast agent. Thus, using PG‐DL reconstruction with a conventional multi‐coil encoding operator leads to analogous signal intensity variations across different time‐frames at the network output, creating difficulties in generalization for varying SNR levels. We propose to use a SIIM encoding operator to capture the signal intensity/SNR variations across time‐frames in a reformulated encoding operator. This leads to a more uniform/flat contrast at the output of the PG‐DL network, facilitating generalizability across time‐frames. PG‐DL reconstruction with the proposed SIIM encoding operator is compared to PG‐DL with conventional encoding operator, split slice‐GRAPPA, locally low‐rank (LLR) regularized reconstruction, low‐rank plus sparse (L + S) reconstruction, and regularized ROCK‐SPIRiT.

**Results:**

Results on highly accelerated free‐breathing first pass myocardial perfusion CMR at three‐fold SMS and four‐fold in‐plane acceleration show that the proposed method improves upon the reconstruction methods use for comparison. Substantial noise reduction is achieved compared to split slice‐GRAPPA, and aliasing artifacts reduction compared to LLR regularized reconstruction, L + S reconstruction and PG‐DL with conventional encoding. Furthermore, a qualitative reader study indicated that proposed method outperformed all methods.

**Conclusion:**

PG‐DL reconstruction with the proposed SIIM encoding operator improves generalization across different time‐frames /SNRs in highly accelerated perfusion CMR.

## 1 INTRODUCTION

Myocardial perfusion cardiac MRI (CMR) is used for functional assessment of stenoses in diagnosing coronary artery disease.^1^, ^2^, ^3^, ^4^, ^5^, ^6^, ^7^ Clinically, myocardial perfusion CMR is acquired using snap‐shot imaging during the first pass of an exogenous contrast agent, which results in limited resolution and coverage.^8^, ^9^, ^10^ Low spatial resolution has been associated with dark rim artifacts that can compromise assessment of perfusion abnormalities.^11^ Additionally, coverage is typically limited to three to four noncontiguous slices,^12^ which may result in missed regions in microvascular disease. Furthermore, limited temporal resolution is associated with low contrast‐to‐noise ratios and may produce cardiac motion artifacts.^13^ Therefore, trade‐offs between spatio‐temporal resolution and coverage still remain a major challenge in myocardial perfusion CMR, necessitating accelerated imaging techniques.

Parallel imaging has long been used in perfusion CMR but is limited to two‐ to three‐fold acceleration.^12^ Spatio‐temporal reconstruction (k‐t) methods^14^, ^15^, ^16^ have been proposed, but their acceleration rates remained limited.^17^ Subsequently, compressed sensing, low‐rank methods, and their combinations have been adopted to perfusion CMR reconstruction to enable higher acceleration rates.^18^, ^19^, ^20^, ^21^, ^22^, ^23^, ^24^, ^25^, ^26^, ^27^, ^28^, ^29^, ^30^, ^31^, ^32^, ^33^, ^34^, ^35^, ^36^, ^37^, ^38^, ^39^, ^40^ These have enabled 3D whole heart myocardial perfusion,^41^, ^42^, ^43^, ^44^, ^45^, ^46^, ^47^, ^48^, ^49^ although a recent study has shown that 2D high resolution scans with smaller temporal footprint are more sensitive for detecting ischemia.^50^ Recently, simultaneous multislice (SMS) imaging has gained interest in CMR for improved coverage with minimal loss in image quality and SNR.^21^, ^23^, ^51^, ^52^, ^53^, ^54^ Yet, ultra‐high acceleration rates are still limited when SMS imaging is combined with in‐plane acceleration due to noise amplification.^55^

Physics‐guided deep learning (PG‐DL) techniques have recently gained substantial interest in accelerated MRI, showing improved reconstruction quality at high acceleration rates compared to parallel imaging or compressed sensing.^56^, ^57^, ^58^, ^59^, ^60^, ^61^, ^62^, ^63^ These PG‐DL techniques use a forward encoding operator incorporating MRI physics, while the proximal operation associated with regularization is solved implicitly by neural networks.^61^ However, PG‐DL networks have several challenges that hamper their applicability in perfusion CMR. A 2D implementation processing slices/time‐frames individually is a natural choice from an implementation perspective, and for avoiding temporal blurring. However, signal intensity changes across time‐frames hinder the utility of such PG‐DL networks, which have exhibited generalizability issues with such variations.^64^ An alternative way to train PG‐DL reconstruction for perfusion CMR would be using a spatio‐temporal network, yet this has its own challenges including memory limitations^65^ and difficulty of procuring high‐quality training databases due to differences in contrast uptakes/breathing patterns among subjects. Thus, application of PG‐DL reconstruction to perfusion CMR has been difficult, and existing DL methods for perfusion CMR reconstruction have been limited to data‐driven image enhancement networks,^66^, ^67^, ^68^ which are trained in a supervised manner using conventional compressed sensing reconstruction outputs as reference images. While this line of work improves reconstruction speed, the reconstruction quality is inherently limited by the conventional reconstruction used as reference for supervised training, which in turn hinders the true potential of DL reconstruction for perfusion CMR.

In this study, we propose to use a signal intensity informed multi‐coil (SIIM) encoding operator in PG‐DL networks to improve highly accelerated perfusion CMR reconstruction. The proposed SIIM encoding operator is inherently aware of contrast/SNR changes across time‐frames, leading to a uniform/flat signal level at the output of the network, which in turn assists the generalizability of PG‐DL methods. Proposed SIIM encoding operator was compared with PG‐DL using conventional operator, and conventional reconstruction methods, including split slice‐GRAPPA,^69^ locally low‐rank (LLR) regularization,^34^, ^70^ regularized ROCK‐SPIRIT^71^ and a low‐rank plus sparse (L + S) reconstruction^35^ for free‐breathing first‐pass perfusion with three‐fold SMS and four‐fold in‐plane acceleration. Results show that PG‐DL reconstruction with the proposed SIIM encoding operator improves upon the other methods by reducing noise and residual artifacts.

## 2 METHODS

### 2.1 PGDL reconstruction

The inverse problem for MRI reconstruction is formulated as an optimization problem

(1) $${\hat{x}}_{\mathrm{reg}}=\arg\min_{x}{\left\|y_{\Omega }-E_{\Omega }x\right\|}_{2}^{2}+R(x),$$

where $y_{\Omega }$ is the acquired multi‐channel *k*‐space, Ω is the in‐plane undersampling pattern, $E_{\Omega }$ is the multi‐coil encoding operator, x is the image of interest, and n is measurement noise. At high acceleration rates, this system is typically ill‐conditioned. The first quadratic term enforces the data fidelity (DF) with acquired k‐space points, and the second term R(·) is a regularizer. This objective function may be solved using a multitude of techniques,^72^ which decouple the DF and regularizer terms into a series of sub‐problems, including variable splitting with quadratic penalty,^61^ described in detail in Supporting Information Figure S1, which is available online.

### 2.2 Conventional multi‐coil encoding operator

The encoding operator $E_{\Omega }$ in Eq. [1] is given as:

(2) $$E_{\Omega }=\left[\begin{array}{c} F_{\Omega }S_{1}\\ \vdots \\ F_{\Omega }S_{C} \end{array}\right],$$

where $F_{\Omega }$ is a sub‐sampled Fourier operator sampling the k‐space locations specified by Ω, and $S_{c}$ is a diagonal matrix representing the $c^{\mathrm{th}}$ coil sensitivity map. In practice, $S_{c}$ are estimated via ESPIRiT,^73^ and inherently encode $B_{1}^{-}$, which remain fixed across time‐frames. Therefore, the solution of Eq. [1] presents varying signal intensities across time‐frames, which mirror SNR variations in acquired k‐space across time‐frames.

### 2.3 SIIM encoding operator

We propose to encode dynamically‐varying signal intensity in the encoding operator for PG‐DL reconstruction. Let L be a diagonal matrix whose entries are the pixel values of an image that contains the signal intensity information of a given time‐frame. We define the SIIM encoding operator as:

(3) $$H_{\Omega }=E_{\Omega }\cdot L,$$

where the inherent signal intensity variation across time‐frames is encoded into encoding operator via L. Note that for perfusion CMR, we indeed have multiple $L^{t},t\in \{1,\cdots ,T\;\}$ where *T* is the number of time‐frames, but for ease of notation, we use L for a given time‐frame of interest. Consequently, the inverse problem for SIIM encoding operator is:

(4) $${\hat{x}}_{\text{SIIM}}=\arg\min_{x}{\left\|y_{\Omega }-H_{\Omega }x\right\|}_{2}^{2}+R(x).$$

In the absence of a regularizer, it is easy to show^74^

(5) $$\begin{array}{ll} {\hat{x}}_{\mathrm{reg}} & ={\left(E_{\Omega }^{*}E_{\Omega }\right)}^{-1}E_{\Omega }^{*}y_{\Omega }\\ & ={\left({\left(L^{-1}\right)}^{*}H_{\Omega }^{*}H_{\Omega }L^{-1}\right)}^{-1}{\left(L^{-1}\right)}^{*}H_{\Omega }^{*}y_{\Omega }\\ & =L{\left(H_{\Omega }^{*}H_{\Omega }\right)}^{-1}H_{\Omega }^{*}y_{\Omega }\\ & =L\cdot {\hat{x}}_{\text{SIIM}}, \end{array}$$

where * is the Hermitian transpose. Thus, the underlying signal intensity information is restored by multiplication with the corresponding signal intensity informed images.

Signal intensity variations for a given time‐frame can be captured with a low‐resolution image, generated from central k‐space, as the diagonal entries of L. In the context of parallel imaging, a similar concept was used, where low‐resolution images from central k‐space were used as coil maps, without normalizing them by their root‐sum‐squares image,^74^ and the signal intensity information was restored by multiplication with the root‐sum‐squares image, as in Eq. [5]. In this work, we instead use the formulation in Eq. [3], since this enables a more synergistic combination with ESPIRiT map estimation.

**FIGURE 1:**
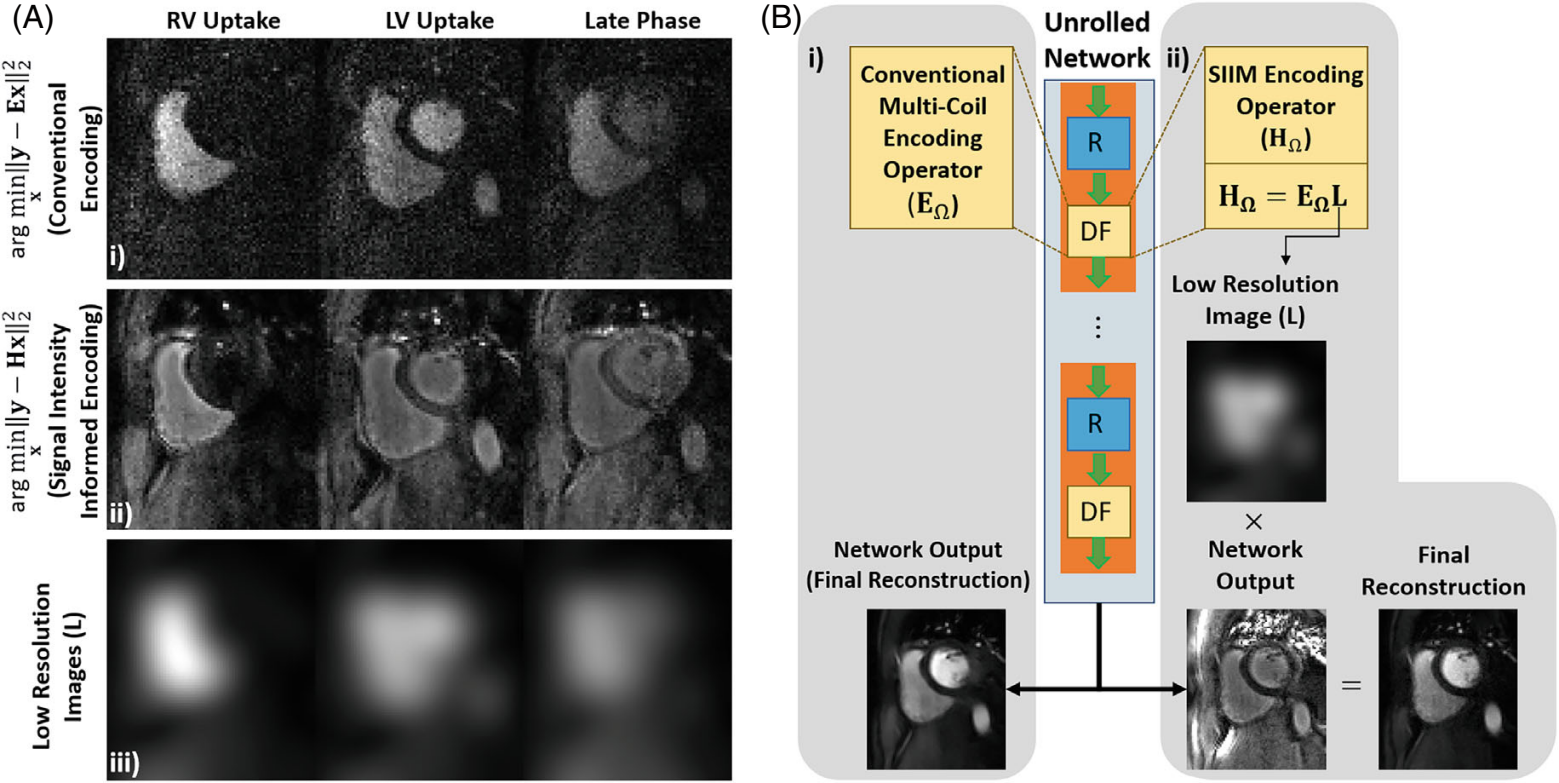
(A), Unregularized least squares estimate of a representative slice using: (i) conventional encoding and (ii) proposed SIIM encoding operator. Signal intensity changes are visible across time‐frames in (i). A more uniform/flat signal level is observed across time‐frames using proposed SIIM encoding operator in (ii). (iii) Corresponding low‐resolution images (L) for the slice of interest for right ventricular uptake (RV), left ventricular uptake (LV), and a late phase are shown. The product of middle and bottom rows yields similar contrast to top row, as given in Eq. [9]. (B), A schematic of PG‐DL reconstruction network with (i) conventional multi‐coil encoding operator using ESPIRiT maps and (ii) proposed SIIM encoding operator. The network outputs are different between the two encoding operators, with the latter showing a flatter signal intensity. The product of the network output for the SIIM operator with low resolution images (L) yields similar contrast to the network output for the conventional operator.

There are two major differences between SIIM and conventional encoding operators. First, there are numerical differences in solving the objective functions in Eqs. [1] and [4], which was also noted for the unregularized case in parallel imaging.^74^ Thus, the SIIM formulation may overcome numerical instabilities at high acceleration rates. Second, in the regularized setup, the SIIM encoding operator has the additional benefit that the solutions of Eq. [4] have more uniform/flat signal intensity across time‐frames of varying SNR, as depicted in Figure 1A. This in turn assists the regularizer to work with consistent signal intensity regardless of the physiological process associated with a time‐frame. Hence, the use of the SIIM operator may lead to improved generalizability for PG‐DL reconstructions, which have been shown to be affected by SNR variations of the underlying solutions. Schematics of unrolled networks using conventional and proposed SIIM encoding operators are shown in Figure 1B.

### 2.4 Imaging experiments

Free‐breathing first‐pass myocardial perfusion CMR was acquired on a 3T Siemens Magnetom Prisma (Siemens Healthineers) in eight subjects (six men, two women, age:39 ± 18 y). This study was approved by our institutional review board, and written informed consent was obtained before each examination. A saturation‐prepared GRE sequence was used, with relevant imaging parameters: FOV = 360 × 320 mm^2^; spatial resolution = 1.7 × 1.7 mm^2^; slice thickness = 8 mm; temporal resolution = 116 ms; SMS factor = 3 (1/3 FOV shifts with CAIPIRINHA [75]); in‐plane acceleration = 4 (uniform undersampling, no ACS) and partial Fourier = 6/8 (overall 16‐fold acceleration).^55^ Non‐prepared GRE calibration scans were acquired at a lower spatial resolution = 1.7 × 5.6 mm^2^ individually for all 9 slices. Details of the imaging sequence are given in Supporting Information Table S1.

### 2.5 SIIM encoding operator formation

Coil maps ($S_{C}$) were generated via ESPIRiT using central 24 × 24 regions of the calibrations scans of the corresponding slices.^73^ Low‐resolution images (L) for each time‐frame and slice were generated from the central 24 × 24 k‐space region reconstructed using split slice‐GRAPPA.^69^ Note that this intermediate reconstruction step was necessary due to the lack of individual k‐spaces for the slices of individual time‐frames resulting from SMS encoding, and would not be necessary for single‐slice/volume imaging. Subsequently, a Blackman filter was applied for ringing,^76^ followed by taking the magnitude of the SENSE‐1 combination of individual coil images.^77^ Finally, SIIM encoding operator $H_{\Omega }$ was generated by multiplying $E_{\Omega }$ by L, whose diagonal entries were the intensity values of the aforementioned magnitude SENSE‐1 image, as in Eq. [3]. Further implementation details for SMS encoding are provided in Supporting Information Figure S2.

### 2.6 Network and training details

Due to lack of fully‐sampled reference data in this highly accelerated SMS perfusion CMR acquisition, the recently proposed self‐supervised learning via data undersampling (SSDU) was used for training.^61^, ^78^, ^79^ Details of the multi‐mask version of SSDU^80^ are given in Supporting Information Figure S3. PG‐DL training with multi‐mask SSDU was performed on 4 subjects using the last 35 time‐frames out of 40, for all three sets of SMS acquisitions per subject for a total number of 420 SMS‐encoded k‐spaces. Training and network details are provided in Supporting Information Table S2 and Figure S4. Two separate trainings were performed using the same network architecture, one with conventional and other with proposed SIIM encoding operator. Implementation of the proposed method will be provided online (https://imagine.umn.edu/research/software).

Testing was performed on 4 different subjects not used in training. Outputs of PG‐DL network with SIIM encoding were multiplied with the corresponding low‐resolution images (L) as shown in Figure 1B to restore the underlying signal intensity. Comparisons were made to split slice‐GRAPPA, LLR regularized reconstruction, L + S reconstruction and regularized ROCK‐SPIRiT, whose hyperparameters were tuned empirically. Further implementation details are provided in Supporting Information Table S3.

Additionally, a numerical perfusion phantom^81^ was used to evaluate the performance of different reconstruction methods, using in‐vivo trained models. The details and results of these numerical experiments are presented in Supporting Information Tables S4 and S5 and Figures S5 and S7.

### 2.7 Image analysis

Qualitative image quality assessment was performed by an experienced cardiologist (15 y of experience). The reader was blinded to the reconstruction methods, orders of which were randomized. Four test subjects (nine slices, all dynamics) were evaluated on a 4‐point ordinal scale, for overall image quality (1:excellent; 2:good; 3:fair; 4:poor), blurring (1:none; 2:mild; 3:moderate; 4:severe) and perceived SNR (1:high SNR; 2:minor noise with moderate SNR; 3:major noise but not limiting clinical diagnosis; 4:poor SNR and nondiagnostic). Wilcoxon signed‐rank test was used to evaluate the scores with a significance level of *P <* 0.05.

## 3 RESULTS

**FIGURE 2:**
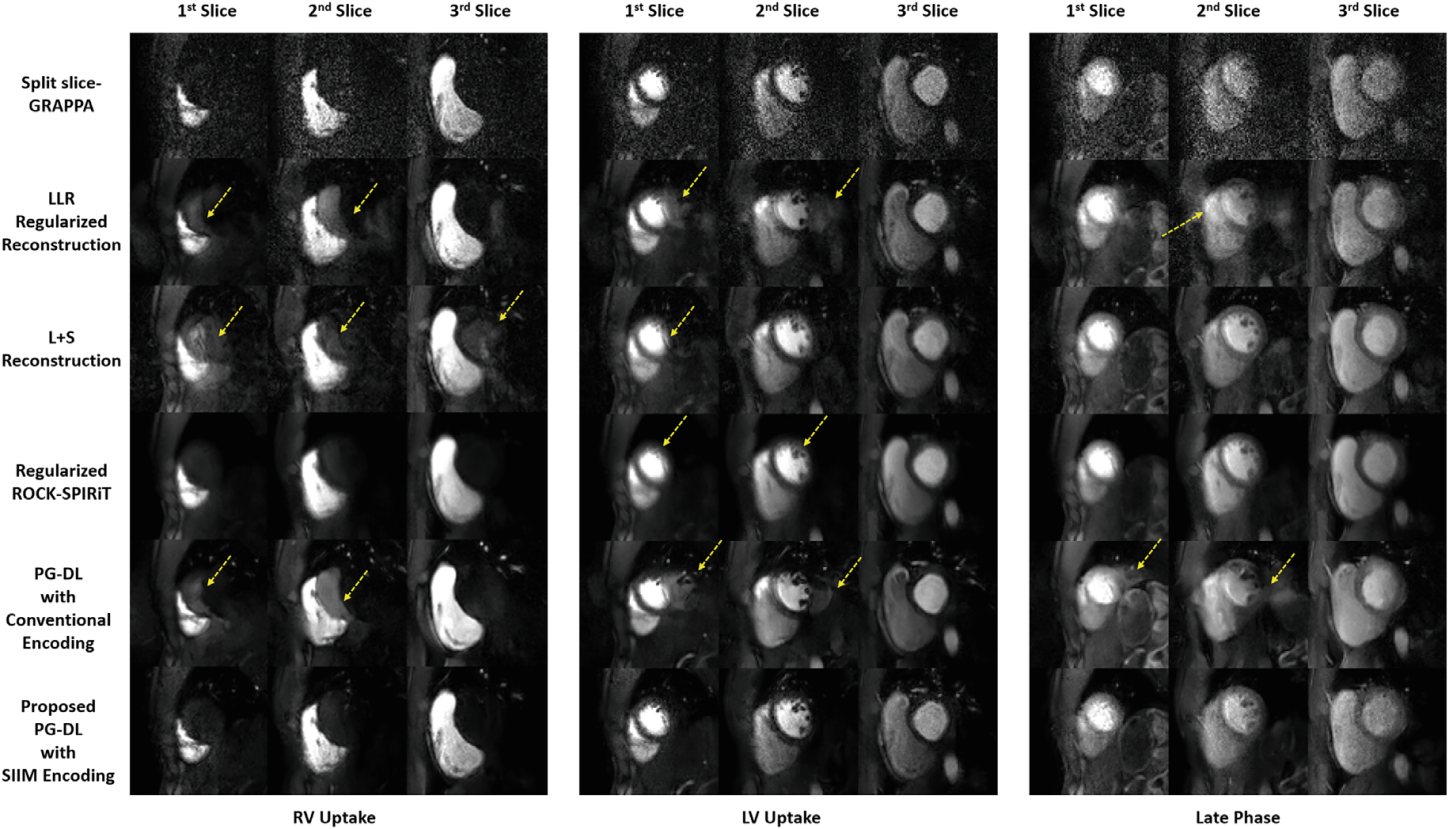
Representative perfusion CMR results across three different time‐frames on a test subject, acquired with a 3‐fold SMS and 4‐fold in‐plane acceleration along with 6/8 partial Fourier (overall 16‐fold acceleration). Split slice‐GRAPPA (top row) shows aliasing‐free reconstruction albeit at substantial noise amplification, while LLR‐regularized reconstruction (second row) and L + S reconstruction (third row) reduce the noise but suffer from aliasing artifacts (yellow arrows). Regularized ROCK‐SPIRiT (fourth row) and PG‐DL with conventional encoding (fifth row) also show reduced noise albeit with blurring and aliasing artifacts (yellow arrows), respectively. Proposed PG‐DL reconstruction with SIIM encoding operator (bottom row) improves upon all techniques showing higher image quality by suppressing noise amplification and aliasing artifacts.

Figure 2 shows reconstructed slices from an SMS slice group for right and left ventricular (RV/LV) uptakes, and a late phase, representing three different signal intensities/SNRs. Split slice‐GRAPPA has no residual aliasing but suffers from high noise amplification especially in late phases with depleted SNR. LLR‐regularized and L + S reconstructions reduce noise amplification, but exhibit residual aliasing artifacts. ROCK‐SPIRiT also reduces noise but suffers from blurring during LV uptake. PG‐DL with conventional encoding operator using ESPIRiT maps has reduced noise, but visible aliasing and inter‐slice leakage. Regularized PG‐DL with proposed SIIM encoding successfully removes aliasing and reduces noise, leading to improved image quality. Difference images between various reconstructions and linear baseline reconstruction split slice‐GRAPPA are depicted in Supporting Information Figure S8. Proposed method shows noise‐like differences with respect to split slice‐GRAPPA, whereas residual artifacts are seen in all other regularized reconstructions. Videos of two subjects are included in Supporting Information Videos [Link], [Link].

**FIGURE 3:**
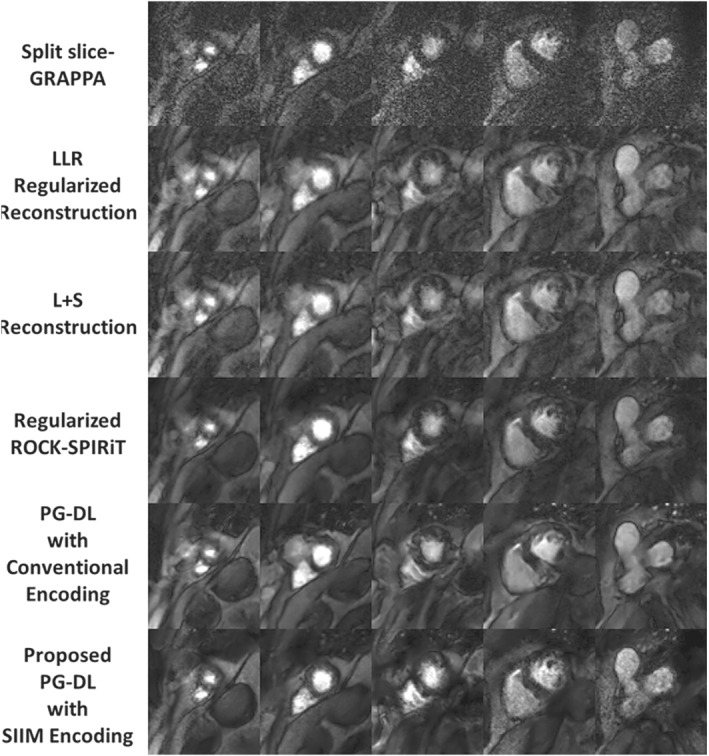
Reconstructions across five slices for a representative time‐frame from another subject. Split slice‐GRAPPA (top row) shows noise amplification across the heart, while LLR‐regularized reconstruction (second row) and L + S reconstruction (third row) have visible residual aliasing artifacts lowering the image quality. On the other hand, regularized ROCK‐SPIRiT (fourth row) shows blurring, and PG‐DL with conventional encoding (fifth row) has visible residual aliasing artifacts especially in the myocardium. Proposed PG‐DL with SIIM encoding operator (bottom row) shows improved image quality with reduced noise and no residual artifacts, as well as a clear delineation of the blood‐myocardium interface. All nine slices using three SMS groups covering the whole heart is depicted in Supporting Information Figure S10.

Figure 3 depicts five slices from a different subject, with all nine slices depicted in Supporting Information Figure S9. Split slice‐GRAPPA shows noise amplification throughout the heart. LLR‐regularized reconstruction, L + S reconstruction and PG‐DL with conventional encoding show reduced noise but visible residual artifacts, while regularized ROCK‐SPIRiT shows blurring. PG‐DL with SIIM encoding eliminates residual aliasing while reducing noise amplification, showing better image quality.

**FIGURE 4:**
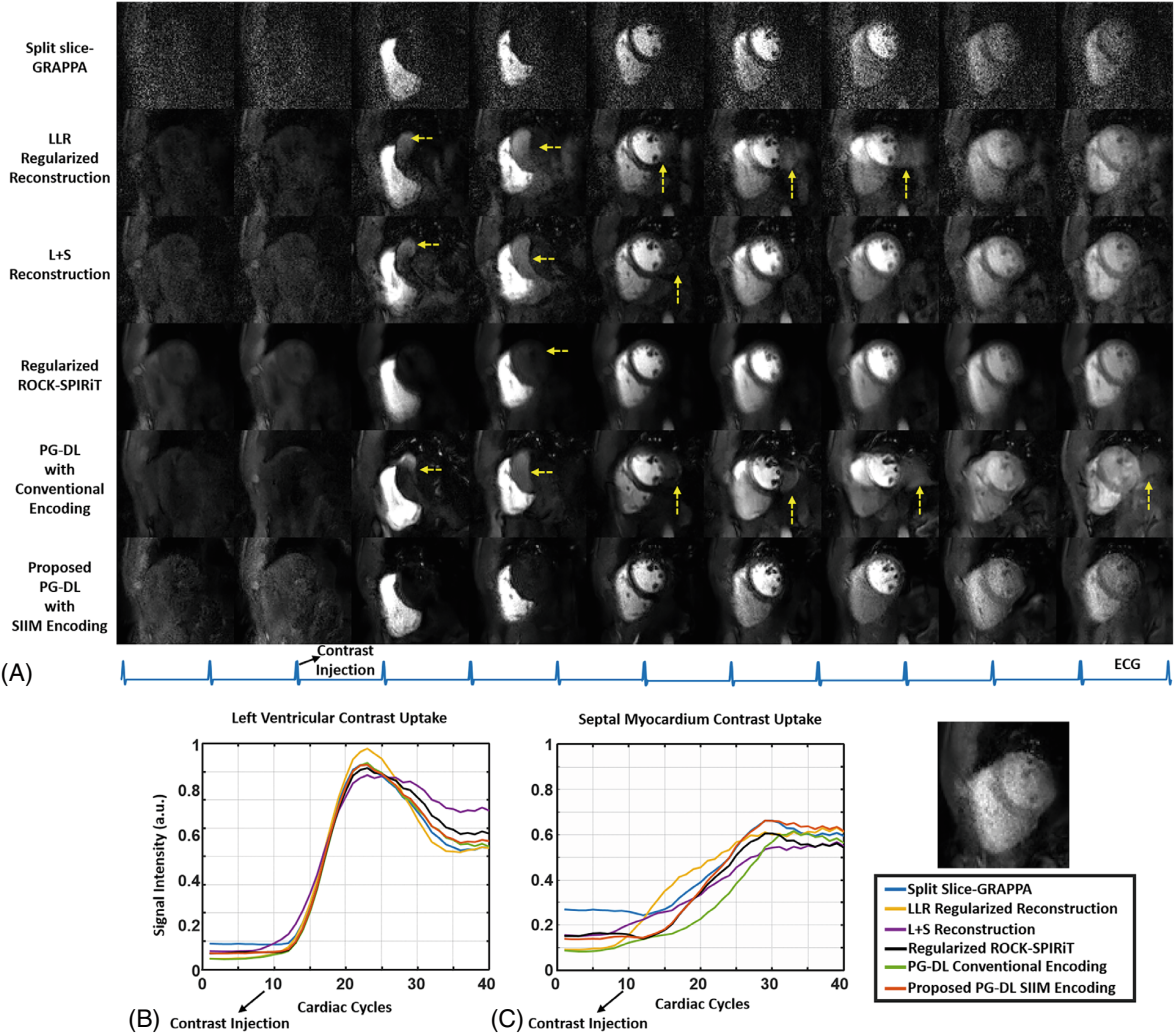
(A), Different time‐frames of a slice of a perfusion CMR scan reconstructed with the different reconstruction methods. Dominant noise amplification is seen across all time‐frames with split slice‐GRAPPA, although no residual aliasing is observed. LLR‐regularized reconstruction, L + S reconstruction and PG‐DL with conventional encoding reduce noise, but suffer from residual inter‐slice aliasing across time‐frames. ROCK‐SPIRiT shows reduced noise with aliasing artifacts in the earlier time‐frames with blurring, especially in left‐ventricular uptake. PG‐DL with SIIM encoding operator shows improved image quality upon all methods, suppressing noise and aliasing artifacts, while maintaining a good image quality. (B), Low‐pass filtered signal intensity curves in the left ventricular blood pool averaged over an ROI in one subject. Split slice‐GRAPPA, regularized ROCK‐SPIRiT, and proposed PG‐DL with SIIM encoding operator show good temporal agreements during contrast uptake, where the averaging across pixels in the ROI reduces the overall effect of noise amplification in the split‐slice GRAPPA curves. (C), Low‐pass filtered signal intensity curves averaged in an ROI in septal myocardium. Split‐slice GRAPPA and proposed PG‐DL with SIIM encoding operator show good temporal agreement in the myocardium uptake curve. The only major differences are observed in the part of the curve prior to contrast injection, where split‐slice GRAPPA exhibits a higher noise floor in the low SNR regime, as expected. On the other hand, PG‐DL with conventional operator, LLR‐regularized reconstruction and L + S reconstruction show severe misestimation of the uptake curve with respect to these two techniques due to dominant residual artifacts in the myocardium. Although regularized ROCK‐SPIRiT follows proposed PG‐DL with SIIM encoding operator during the first half of the time‐frames, it drifts away in later phases due to blurring that distorts the myocardium blood‐interface.

Figure 4A shows different time‐frames of a slice. Dominant noise amplification is seen with split slice‐GRAPPA, but without residual aliasing. LLR‐regularized reconstruction, L + S reconstruction and PG‐DL with conventional encoding reduce noise, but suffer from residual inter‐slice aliasing. Similarly regularized ROCK‐SPIRiT shows reduced noise, but exhibits blurring in LV uptake and aliasing in earlier time‐frames. PG‐DL with SIIM encoding shows improved image quality, suppressing noise and aliasing artifacts. Figure 4B,C show low‐pass filtered^82^ signal intensity curves in the LV blood pool and septal myocardium, averaged over respective ROIs. Split‐slice GRAPPA and proposed method show good temporal agreement in myocardial uptake, with differences only prior to contrast injection, where split‐slice GRAPPA exhibits a higher noise floor in the low SNR regime, as expected. The other reconstructions show misestimation of the uptake curve due to residual and blurring artifacts, consistent with earlier results. Myocardial signal intensity curves for six AHA sectors are shown in Supporting Information Figure S10.

**FIGURE 5:**
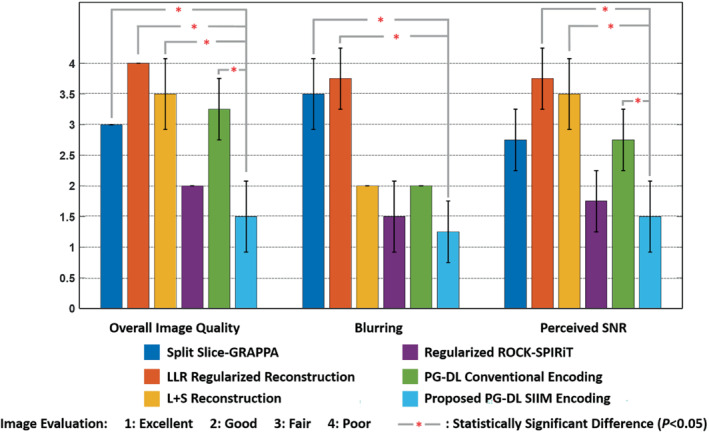
The clinical reader study results for the test data sets. Bar plots show average reader scores and their SD across the test subjects for all six reconstruction methods. Statistical testing was performed using Wilcoxon signed‐rank test, and * shows significant statistical difference with *P* < 0.05. The proposed PG‐DL with SIIM encoding shows the highest overall image quality scores among all methods with significant improvement upon split‐slice GRAPPA, LLR‐regularized reconstruction, L + S reconstruction and PG‐DL with conventional encoding. The proposed method also has the least amount of blurring among all methods, and significantly improves upon split slice‐GRAPPA and LLR‐regularized reconstruction in terms of blurring. Among all methods, LLR‐regularized reconstruction, L + S reconstruction and PG‐DL with conventional encoding show the least amount of perceived SNR, whereas the proposed method shows the highest perceived SNR meanwhile significantly improved upon them.

Figure 5 summarizes the reader study. Proposed PG‐DL with SIIM encoding operator shows the best overall image quality among the methods, significantly improving on split slice‐GRAPPA, LLR‐regularized reconstruction, L + S reconstruction and PG‐DL with conventional encoding. Similarly, the proposed method shows the least amount of blurring and highest perceived SNR among all methods.

## 4 DISCUSSION

In this study, we proposed SIIM encoding operator for PG‐DL reconstruction of image series with varying contrast across time‐frames, and applied it to highly accelerated myocardial perfusion CMR. The main advantage of using SIIM encoding operator is a uniform/flat signal level across different time‐frames at the unrolled neural network output. This in turn facilitates generalizability of PG‐DL reconstruction. The proposed approach improved upon multiple regularized reconstructions, showing better image quality, and reduced noise amplification and aliasing.

Conventional and SIIM encoding have two main differences. First, the solution of Eq. [1] using conventional encoding is adversely affected by ill‐conditioning at high accelerations.^83^ As noted earlier, a similar concept to SIIM encoding was proposed in^74^ to improve numerical stability for parallel imaging. The proposed SIIM encoding in Eq. [3] aims for a similar improvement, while enabling a synergistic combination with ESPIRiT, thus not necessitating a different coil map generation process as in.^74^ Second, and more importantly, in the PG‐DL setup, SIIM encoding provides a more uniform contrast at the neural network outputs across time‐frames. The output signal intensity of PG‐DL with conventional encoding operator fluctuates across time‐frames, and the regularization in the unrolled network needs to work with dramatically different signal levels. On the other hand, SIIM encoding operator maintains a uniform output in terms of signal level. Thus, regularization operates on more uniform SNRs in image space, for the corresponding outputs ${\hat{x}}_{\text{SIIM}}$, which empirically generalizes better across time‐frames. Even though this intermediate solution has more uniform signal intensity, the final reconstruction is generated by multiplying with the corresponding low‐resolution image for that time‐frame, restoring the original signal intensity, as indicated in Eq. [2]. Thus, the use of SIIM encoding operator should not affect quantification in myocardial perfusion, consistent with conclusions from the uptake curves. Finally, on a first look, Eq. [5] may resemble preconditioners in other MRI reconstruction problems,^84^, ^85^, ^86^ which are used to reduce the number of iterations for data fidelity. However, such preconditioners do not change the output signal intensity, thus solution of the preconditioned system coincides with that of the objective in Eq. [1]. Hence, SIIM operator is distinct from this typical use of preconditioning, leading to a more uniform signal intensity across time‐frames.

DL reconstruction has gained interest in perfusion CMR, but has been limited to data‐driven image enhancement approaches that learn a mapping between aliased and artifact‐free images.^66^, ^67^, ^68^ PG‐DL approaches, which have been shown to outperform image enhancement methods^62^, ^87^, ^88^ have remained elusive for perfusion CMR. One of the main challenges for PG‐DL techniques has been related to generalizability with SNR changes,^64^ limiting the use of such reconstructions across perfusion time‐frames, which is the main issue tackled in this study. Another challenge for DL reconstruction in perfusion CMR has been the lack of gold‐standard reference data. Aforementioned data‐driven DL methods^66^, ^67^, ^68^ were trained using supervision with compressed sensing reconstructions, limiting the performance of DL reconstruction. On the other hand, PG‐DL methods enable self‐supervised training from undersampled k‐space data only,^61^, ^78^, ^80^, ^89^, ^90^ without a reference image. Thus, the combination of SIIM encoding and self‐supervised learning for PG‐DL, as in this study, has the potential to further improve the utility of DL reconstruction for perfusion CMR.^61^ Finally, PG‐DL reconstruction can be trained with fewer datasets compared to data‐driven DL methods, and the number of k‐spaces used for training in this work was in line with earlier PG‐DL works that used ∼200‐to‐360 k‐spaces,^56^, ^57^, ^58^, ^59^, ^60^, ^61^, ^64^, ^91^, ^92^ and was gathered using only four subjects. We note that the performance gap between the DL methods may change with a substantially larger training database, but this could not be investigated with our current cohort size.

The use of SMS encoding in this study required several design choices related to calibration data. First, since central k‐space data were not available for individual slices for each time‐frame, an initial split slice‐GRAPPA reconstruction was used to generate **L**, which suppresses aliasing but shows noise amplification. However, since only a limited central k‐space region, containing high‐SNR low‐frequency k‐space points, was used to generate **L**, SNR reduction effects from split slice‐GRAPPA were observed to be minimal in subsequent processing. We emphasize that this step was only needed because of SMS encoding, and is not necessary for conventional 2D/3D encoding, where central k‐space can be fully‐sampled. Furthermore, Blackman filtering was used to avoid ringing, and the reader study did not report any dark rim artifacts associated with the use of **L**. Second, calibration data for SMS reconstruction were acquired separately in free‐breathing, which may be in different respiratory/cardiac motion states than perfusion data. Previously, it was shown that there were no differences between using free‐breathing and breath‐held calibration in another SMS CMR application in healthy cohorts.^93^ Furthermore, ESPIRiT uses only a 24 × 24 central region, leading to smooth maps, where motion‐related artifacts in coil estimation may be non‐severe for most cohorts. However, evaluation of these pre‐acquired calibration scans warrants further investigation, especially in patient populations with pharmacologically induced stress. Finally, uniform undersampling was used in combination with SMS, since it allows easier integration in clinical sequences, and enables comparisons with clinically‐used split slice‐GRAPPA reconstruction. We note that compressed sensing methods are often used with random undersampling, thus their performance with uniform undersampling may be deteriorated.

This study has several limitations. All acquisitions in this study were prospectively accelerated. Therefore, there is no gold‐standard reference for image quality assessment. Since it is difficult to acquire first‐pass perfusion on subjects multiple times due to need for repeated contrast injection, a conventional low‐resolution perfusion scan with limited coverage was not performed, excluding a more typical clinical baseline for comparison. Additionally, no stress imaging data were available, which is clinically imperative for perfusion diagnostics. A pixel‐wise mapping of myocardial blood flow (MBF) estimation^94^ may be performed for quantitative assessment,^95^ but such analyses typically require modifications to the imaging protocol, such as administering dual doses^96^, ^97^ or using dual sequences.^98^, ^99^, ^100^, ^101^ Thus, MBF estimation could not be reliably performed with our acquisition protocol. Further clinical studies are warranted to assess full potential of the proposed method, and its diagnostic value in patients with suspected coronary artery disease.

## 5 CONCLUSIONS

The proposed PG‐DL reconstruction with SIIM encoding operator generalizes well across time‐frames/SNRs, and substantially improves upon several existing reconstruction methods for highly accelerated perfusion CMR.

### FUNDING INFROMATION

NIH, Grant numbers: R01HL153146, R21EB028369, P41EB027061; NSF, Grant number: CAREER CCF‐1651825; NWO Start‐Up Grant STU.019.024, 4TU Federation, Health Technology Programme TU Delft ‐ LUMC; AHA Predoctoral Fellowship.

## Supporting information


**Video S1.** Movie of the perfusion images reconstructed with all techniques of a test subject (shown in Figure 2).

**Video S2.** Movie of the perfusion images reconstructed with all techniques of another test subject.

**Figure S1.** Details of variable splitting for solving the inverse problem.
**Figure S2.** Details of implementation for SMS encoding (A) SMS forward model for the acquisition where $y_{\Omega }^{\mathrm{SMS}}$ is the acquired multi‐channel SMS k‐space, $E_{\Omega }^{\left[i\right]}$ is the multi‐coil encoding operator of the *i*th slice, $x^{\left[i\right]}$ is the underlying image corresponding to the i^th^ simultaneously excited slice, and $n_{\text{slice}}$ is the number of SMS‐excited slices. (B) The notation in (A) can be condensed by concatenating simultaneously excited slices, ${\left\{x^{\left[i\right]}\right\}}_{i=1}^{n_{\text{slice}}}$, along the readout direction, as $x^{\mathrm{SMS}}$ which yields a compact form of multi‐coil and multi‐slice operator $E_{\Omega }^{\mathrm{SMS}}=\left[E_{\Omega }^{\left[1\right]}\;\cdots \;E_{\Omega }^{\left[n_{\text{slice}}\right]}\right]$.^5^, ^6^, ^7^ (C) For the SIIM encoding operator, we let $L_{\mathrm{SMS}}$ be a block diagonal matrix whose entries are also diagonal matrices, $L_{\left[i\right]}$ that encode the signal variations in the *i*th SMS‐excited slice, such as a low‐resolution image, defined as $L_{\mathrm{SMS}}$. Note that, as before, for ease of notation, we simply use $L_{\left[i\right]}$, but there are $T\cdot n_{\text{slice}}$ different low‐resolution images with $L_{\left[i\right]}^{t},t\in \left\{1,\cdots ,T\;\right\},i\in \left\{1,\cdots ,n_{\text{slice}}\;\right\},$ when different time‐frames are considered. Finally, SIIM encoding operator for SMS imaging is given as $H_{\Omega }^{\mathrm{SMS}}=E_{\Omega }^{\mathrm{SMS}}L_{\mathrm{SMS}}$
**Figure S3.** Details of self‐supervised deep learning implementation
**Figure S4.** (A) The ResNet structure consisted of 15 residual blocks with skip connections which were used to facilitate the information flow during training and each block has two convolutional layers.^11^ (A) The ResNet structure consisted of 15 residual blocks with skip connections which were used to facilitate the information flow during training and each block has two convolutional layers.^9^ (B) First layer of the residual block was followed by a rectified linear unit (ReLU) and the latter was followed by a constant multiplication corresponding to 0.1.^9^ All layers in the network had kernel size of 3 × 3 and 64 channels, for a total of 592 129 trainable parameters which were shared across unrolled iterations. The three SMS slices were concatenated along the readout direction prior to being input to the ResNet with proper FOV shifts to reorient the CAIPIRINHA phase cycling and avoid boundary artifacts.^5^, ^6^
**Figure S5.** Representative numerical phantom results for myocardial perfusion CMR simulated with a 3‐fold SMS and 4‐fold in‐plane acceleration along with outer volume suppression.
**Figure S6.** The error images between full‐sampled reference images and all reconstruction methods for the numerical phantom experiments.
**Figure S7.** Signal intensity plots of left ventricular contrast uptake and 6 AHA segments of myocardial contrast uptake for all methods.
**Figure S8.** Difference images between split slice‐GRAPPA and all the regularized reconstruction methods.
**Figure S9.** All 9 slices for a representative time‐frame, covering the whole heart using 3 SMS groups of 9 slices from another subject shown in Figure 3.
**Figure S10.** Low‐pass filtered signal intensity curves of hand‐drawn 6 AHA sectors in myocardium.
**Table S1.** Free‐breathing first‐pass myocardial perfusion CMR imaging sequence details.^4^
**Table S2.** Implementation details of the PG‐DL networks used in this study.
**Table S3.** Implementation details of comparison reconstruction methods. All thresholding values were empirically tuned to maximize visual image quality.
**Table S4.** Implementation details of the numerical phantom.
**Table S5.** Image quality assessment scores using PSNR and SSIM for all reconstruction methods using the numerical phantom.
