# Supplementary material for: Signal intensity informed multi‐coil encoding operator for physics‐guided deep learning reconstruction of highly accelerated myocardial perfusion CMR
Source: Magn Reson Med. 2022 Sep 21;89(1):308–21. doi: 10.1002/mrm.29453 (PMC9617789; doi:10.1002/mrm.29453)
Supplement: Supplementary file 3 — Figure S1. Details of variable splitting for solving the inverse problem. Figure S2. Details of implementation for SMS encoding (A) SMS forward model for the acquisition where yΩSMS is the acquired multi‐channel SMS k‐space, EΩ[i] is the multi‐coil encoding operator of the ith slice, x[i] is the underlying image corresponding to the ith simultaneously excited slice, and nslice is the number of SMS‐excited slices. (B) The notation in (A) can be condensed by concatenating simultaneously excited slices, x[i]i=1nslice, along the readout direction, as xSMS which yields a compact form of multi‐coil and multi‐slice operator EΩSMS=EΩ[1]⋯EΩnslice. 5 , 6 , 7 (C) For the SIIM encoding operator, we let LSMS be a block diagonal matrix whose entries are also diagonal matrices, L[i] that encode the signal variations in the ith SMS‐excited slice, such as a low‐resolution image, defined as LSMS. Note that, as before, for ease of notation, we simply use L[i], but there are T·nslice different low‐resolution images with L[i]t,t∈{1,⋯,T},i∈1,⋯,nslice, when different time‐frames are considered. Finally, SIIM encoding operator for SMS imaging is given as HΩSMS=EΩSMSLSMS Figure S3. Details of self‐supervised deep learning implementation Figure S4. (A) The ResNet structure consisted of 15 residual blocks with skip connections which were used to facilitate the information flow during training and each block has two convolutional layers. 11 (A) The ResNet structure consisted of 15 residual blocks with skip connections which were used to facilitate the information flow during training and each block has two convolutional layers. 9 (B) First layer of the residual block was followed by a rectified linear unit (ReLU) and the latter was followed by a constant multiplication corresponding to 0.1. 9 All layers in the network had kernel size of 3 × 3 and 64 channels, for a total of 592 129 trainable parameters which were shared across unrolled iterations. The three SMS slices were concatenated along t [file MRM-89-308-s003.docx]

**Supporting Information**

1. **Details of the Implementations**

**
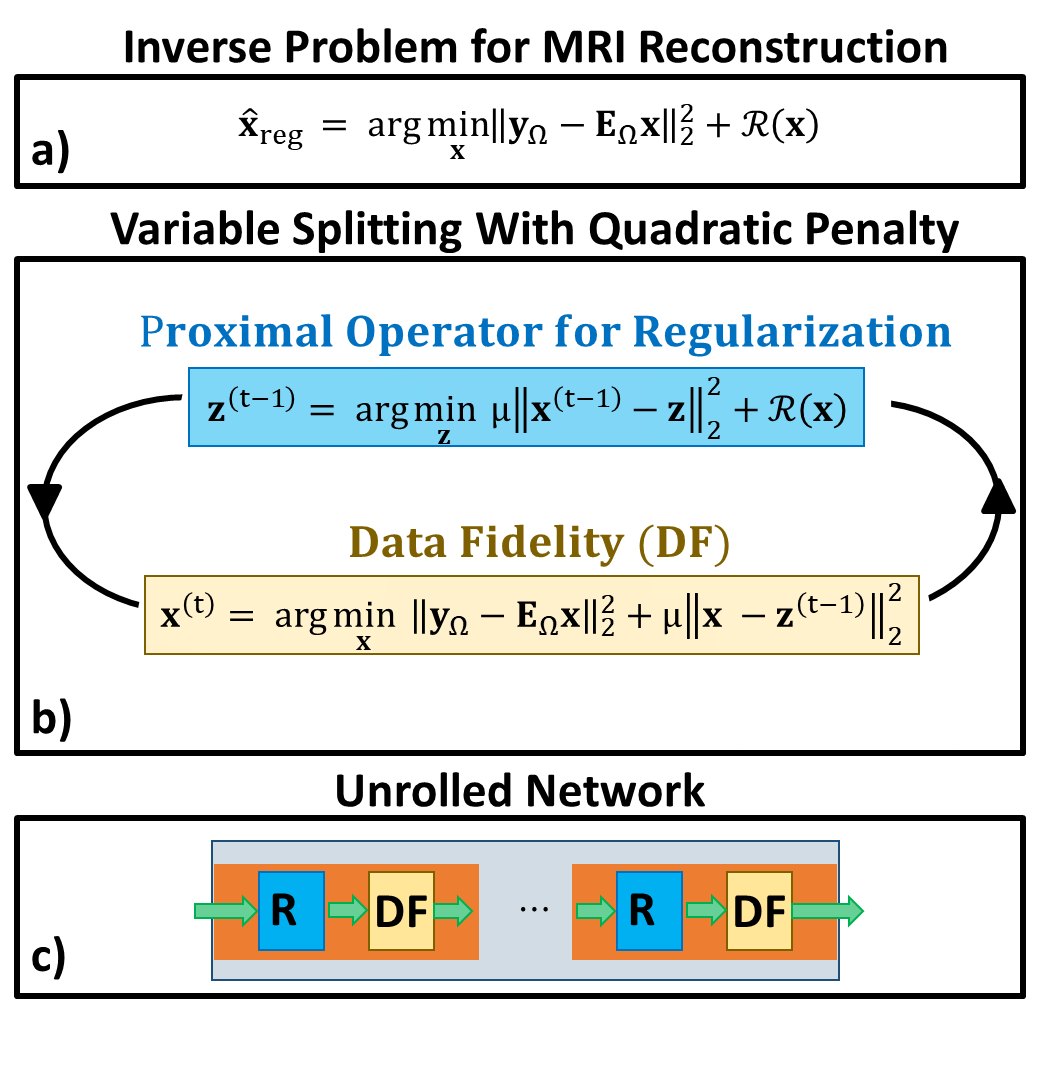
**

**Supporting Figure S1:** Details of variable splitting for solving the inverse problem. **(a)** The inverse problem for MRI reconstruction given in Eq. [1] of the main text. **(b)** Equation in (a) can be solved using multitude of techniques (1), which decouple the data fidelity (DF) and regularizer terms into a series of sub-problems, including variable splitting with quadratic penalty (2), shown here, where $\mathbf{z}^{(t)}$ is an auxiliary variable at $t^{\mathrm{th}}$ iteration, $\mathbf{x}^{(t)}$ is the reconstructed image at $t^{\mathrm{th}}$ iteration and $\mu$ is the quadratic penalty parameter. **(c)** In PG-DL methods, the decoupled problem in (b) is unrolled for a fixed number of iterations, where the proximal operation for the regularization is typically solved using a neural network, and the DF subproblem is solved via conventional linear methods, such as conjugate gradient (2,3).

| **Contrast Agent** | 0.05 mmol/kg gadabutrol (Gadovist) |
| --- | --- |
| **Contrast Amount** | 4mL/s followed by a 10 mL saline flush |
| **Acquisition** | Saturation-prepared GRE |
| **TR** | 2.9 ms |
| **TE** | 1.7 ms |
| **Flip Angle** | 12° |
| **FOV** | 360 ×320 mm^2^ |
| **Spatial Resolution** | 1.7 × 1.7 mm^2^ |
| **Slice Thickness** | 8 mm |
| **Temporal Resolution** | 116 ms |
| **Saturation Time** | 150 ms |
| **SMS Factor** | 3 |
| **Number of SMS Sets** | 3 |
| **In-plane Acceleration** | 4 (no ACS) |
| **Partial Fourier** | ON (6/8) |
| **Total Acceleration** | 16 |
| **Outer Volume Suppression (OVS)** | ON – Slab Selective |
| **OVS RF** | 3.8 ms asymmetric sinc |
| **OVS RF Peak Shift** | 15% |
| **OVS Saturation Slab** | 150 mm (each side) |
| **OVS Time-bandwidth Product (TBP)** | 8 |
| **CAIPIRINHA Shifts** | 2π/3 (1/3 FOV) |

**Supporting Table S1:** Free-breathing first-pass myocardial perfusion CMR imaging sequence details (4). Three sets of SMS-accelerated slices were acquired for a total of 9 slices, covering the whole heart where OVS modules were interleaved between every 9 imaging pulses to maintain suppression throughout the imaging (4). A non-prepared GRE was used to acquire calibration scans with FOV = 360 × 360 mm^2^ and at a lower spatial resolution = 1.7 × 5.6 mm^2^ individually for all 9 slices in free-breathing.

**
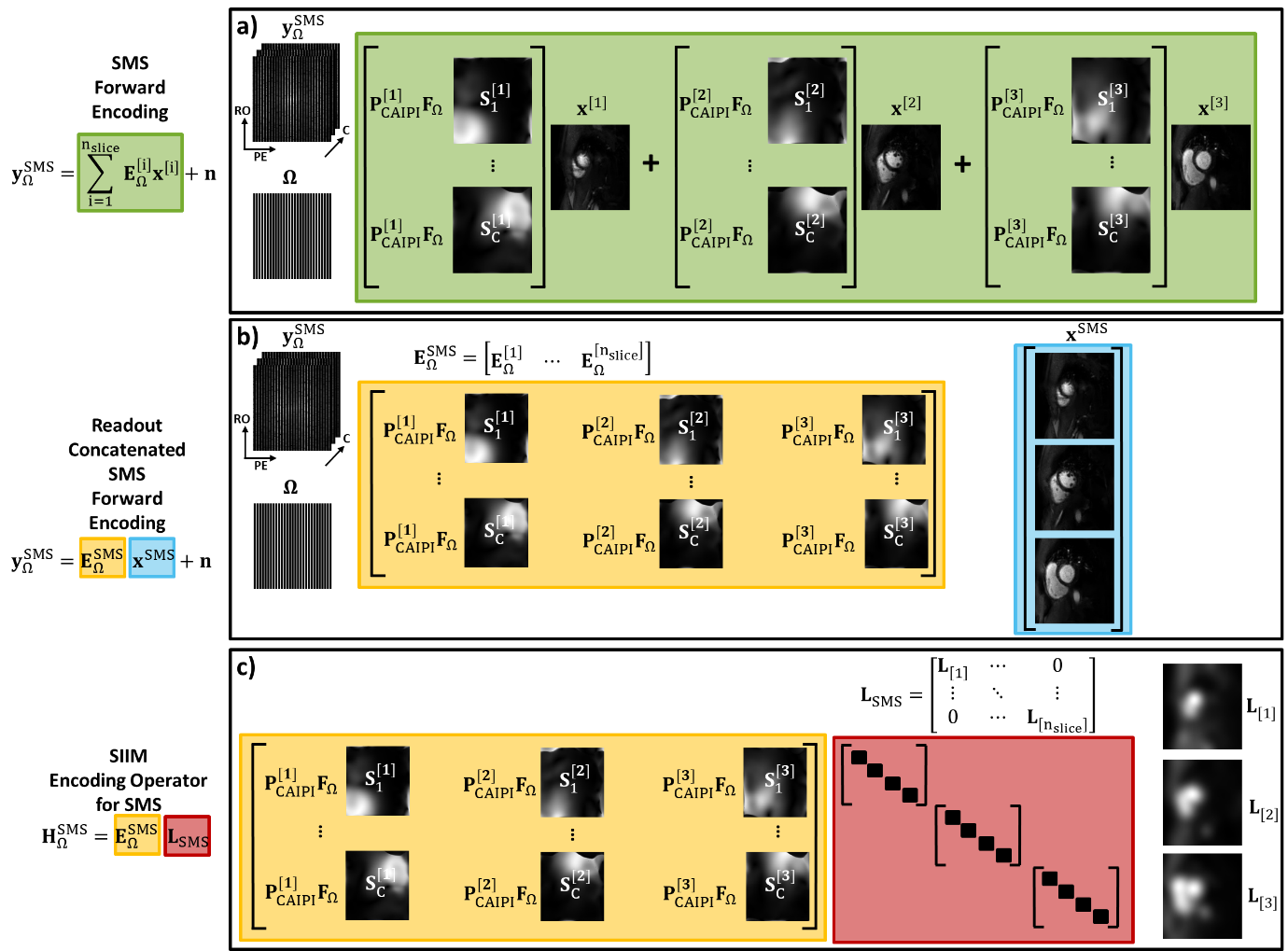
**

**Supporting Figure S2:** Details of implementation for SMS encoding **(a)** SMS forward model for the acquisition where $\mathbf{y}_{\Omega}^{\mathrm{SMS}}$ is the acquired multi-channel SMS k-space, $\mathbf{E}_{\Omega}^{[i]}$ is the multi-coil encoding operator of the i^th^ slice, $\mathbf{x}^{[i]}$ is the underlying image corresponding to the i^th^ simultaneously excited slice, and $n_{\mathrm{slice}}$ is the number of SMS-excited slices. **(b)** The notation in (a) can be condensed by concatenating simultaneously excited slices, $\left\{ \mathbf{x}^{[i]} \right\}_{i=1}^{n_{\mathrm{slice}}}\boldsymbol{,}$ along the readout direction, as $\mathbf{x}^{\mathrm{SMS}}$ which yields a compact form of multi-coil and multi-slice operator $\mathbf{E}_{\Omega}^{\mathrm{SMS}}=\left[ \begin{matrix} \mathbf{E}_{\Omega}^{[1]} & \boldsymbol{\cdots} & \mathbf{E}_{\Omega}^{{[n}_{\mathrm{slice}}]} \end{matrix} \right]$ (5-7). **(c)** For the SIIM encoding operator, we let $\mathbf{L}_{\mathrm{SMS}}$ be a block diagonal matrix whose entries are also diagonal matrices, $\mathbf{L}_{[i]}$ that encode the signal variations in the i^th^ SMS-excited slice, such as a low-resolution image, defined as $\mathbf{L}_{\mathrm{SMS}}$. Note that, as before, for ease of notation, we simply use $\mathbf{L}_{[i]}$**,** but there are $T\cdot n_{\mathrm{slice}}$ different low-resolution images with $\mathbf{L}_{\left[ i \right]}^{t} , t \in\left\{ 1,\cdots,T \right\},i \in\left\{ 1,\cdots,n_{\mathrm{slice}} \right\},$ when different time-frames are considered. Finally, SIIM encoding operator for SMS imaging is given as $\mathbf{H}_{\Omega}^{\mathrm{SMS}}=\mathbf{E}_{\Omega}^{\mathrm{SMS}}\mathbf{L}_{\mathrm{SMS}}.$

**
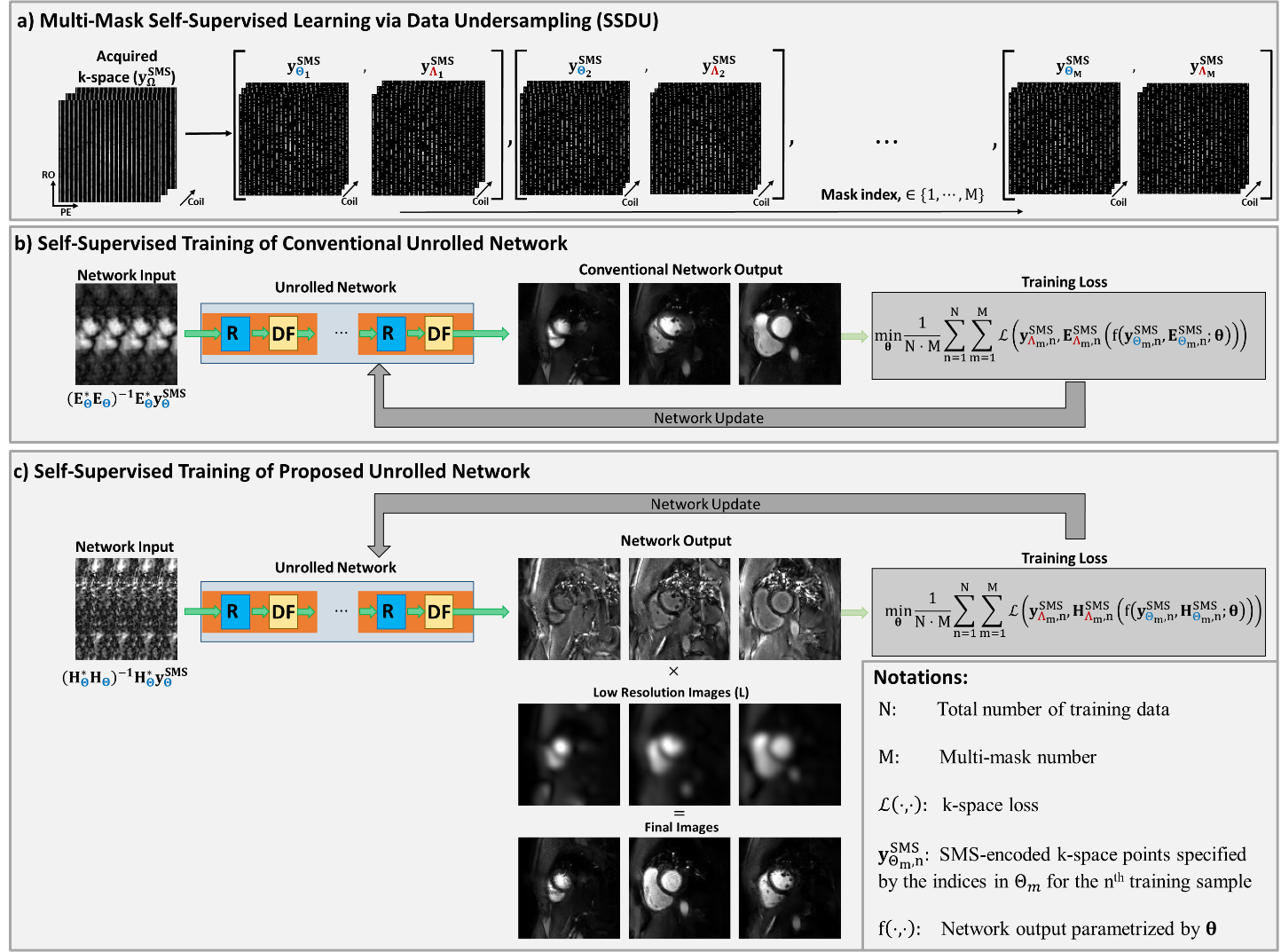
**

**Supporting Figure S3:** Details of self-supervised deep learning implementation. **(a)** SSDU splits the acquired k-space locations $\Omega$ into two disjoint sets, $\Theta$ and $\Lambda$, where the former is used to enforce DF in the unrolled network, and the latter is used to define k-space loss (2). Multi-mask SSDU (8), used in this work, utilizes multiple disjoint sets i.e. $\Omega=\Theta_{m} \bigcup\Lambda_{m}, m\in\{1,\cdots,M\}$ to further improve reconstruction quality, **(b)** Self-supervised training of conventional unrolled network is depicted along with the training loss function where The network output is mapped back to an unseen part of the acquired k-space using the encoding operator $\mathbf{E}_{\Lambda_{m},n}^{\mathrm{SMS}}$ for the$k^{\mathrm{th}}$ m^th^ loss mask $\Lambda_{m}$ and $n^{\mathrm{th}}$ training data sample. **(c)** Self-supervised training of proposed unrolled network is depicted with the encoding operator $\mathbf{H}_{\Lambda_{m},n}^{\mathrm{SMS}}$ for the$k^{\mathrm{th}}$ m^th^ loss mask $\Lambda_{m}$ and $n^{\mathrm{th}}$ training data sample. Note that the training loss equations in (a) and (b), loss is calculated between the corresponding unseen k-space points defined $\mathbf{y}_{\Lambda_{m},n}^{\mathrm{SMS}}$ and the output of the unrolled network that is taken back to k-space. In the conventional multi-coil operator case, this leads to the use of $\mathbf{E}_{\Theta_{m},n}^{\mathrm{SMS}}$ as the input to the network, or equivalently as the second argument of $f(\cdot,\cdot)$ that describes the network output, and $\mathbf{E}_{\Lambda_{m},n}^{\mathrm{SMS}}$as the operator that takes the network output to the k-space. Analogoulsy, for the proposed SIIM operator setting, we use $\mathbf{H}_{\Theta_{m},n}^{\mathrm{SMS}}$ as the input to the network along with $\mathbf{H}_{\Lambda_{m},n}^{\mathrm{SMS}}$ as the operator that takes the output of the network to the k-space. Note that this difference corresponds to the use of different forward encoding operator in the objective functions for these models, as specified in Eq. [1] and [4], respectively.

| **Total number of unrolls/cascades** | 10 |
| --- | --- |
| **Data fidelity unit** | Conjugate Gradient (20 iterations) |
| **Proximal operator** | ResNet (Supporting Figure 4) |
| **ESPIRiT ACS region size** | 24×24 |
| **ESPIRiT Calibration Kernel** | 6×6 |
| **ESPIRiT Threshold** | 0.02 |
| **Software Library** | *TensorFlow* |
| **GPU** | NVIDIA Tesla V100 (32 GB memory) |
| **CPU** | Intel(R) Xeon(R) Gold 6136 |
| **Optimization Algorithm** | Adam optimizer |
| **Learning Rate** | 3 ∙ 10^-4^ |
| **Loss** | Normalized $\mathcal{l}_{1}-\mathcal{l}_{2}$ loss |
| **Number of Epochs** | 100 |
| **Number of Trainable Parameters** | 592,129 |

**Supporting Table S2:** Implementation details of the PG-DL networks used in this study. The ESPIRiT coil maps were used within the encoding operator in DF for conventional and SIIM encoding operator. A convolutional neural network (CNN) based on a ResNet structure (9) shown in **Supporting Figure S4**, which has shown success in other regression problems (9), was used to solve the proximal operation in the regularization sub-problem shown in **Supporting Figure S1.** For the DF sub-problem, a simple generic preconditioner with diagonal entries (10) was used for each slice, where an approximation of the diagonal entries of the related encoding operator for uniform undersampling is calculated by $\sum_{c=1}^{C} \left( \mathbf{S}_{c}\mathbf{LLS}_{c}^{*} \right)^{-1}/ \left( \left| \Omega\right|/\left( n_{\mathrm{RO}}\cdot n_{\mathrm{PE}} \right) \right),$ where $n_{RO}$ and $n_{PE}$ being the image sizes, and $C$the number of channels. Note that this preconditioner amounts to a constant multiple of the identity matrix in the conventional encoding operator setting, reducing to the conventional CG algorithm.


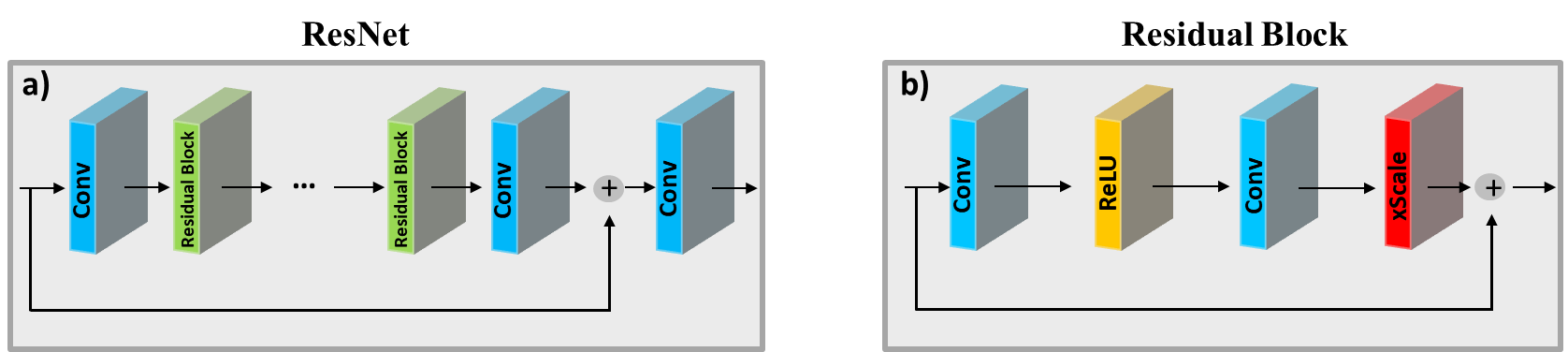


**Supporting Figure S4: (a)** The ResNet structure consisted of 15 residual blocks with skip connections which were used to facilitate the information flow during training and each block has two convolutional layers (9). **(b)** First layer of the residual block was followed by a rectified linear unit (ReLU) and the latter was followed by a constant multiplication corresponding to 0.1 (9). All layers in the network had kernel size of 3 × 3 and 64 channels, for a total of 592,129 trainable parameters which were shared across unrolled iterations. The three SMS slices were concatenated along the readout direction prior to being input to the ResNet with proper FOV shifts to reorient the CAIPIRINHA phase cycling and avoid boundary artifacts (5,6).

| **Reconstruction Method** | **Calibration**  **Kernel Size** | **Regularizer Details** |
| --- | --- | --- |
| Split Slice-GRAPPA | 7×7 (SMS) 7×6 (In-plane) | N/A |
| LLR Regularized Reconstruction | N/A | Thresholding: 0.1 × the $\mathcal{l}_{\infty}$ norm of the zero-filled SMS image (run on 8×8 pixel blocks) |
| L+S Reconstruction | N/A | SVD: 0.01 × the highest singular value of the zero-filled SMS  Sparsity: 0.01 × the $\mathcal{l}_{\infty}$ norm of the zero-filled SMS image |
| ROCK-SPIRiT | 9×9 | Thresholding: 0.075 × the $\mathcal{l}_{\infty}$ norm of the zero-filled SMS image (run on 8×8 pixel blocks) |

**Supporting Table S3:** Implementation details of comparison reconstruction methods. All thresholding values were empirically tuned to maximize visual image quality.

1. **Numerical Phantom Experiments**

| **Respiratory Motion** | ON |
| --- | --- |
| **Number of Coils** | 34 |
| **Coil Distance** | 350 mm (distance of coil centers from origin) |
| **SMS Factor** | 3 |
| **In-plane Acceleration** | 4 (no ACS) |
| **Partial Fourier** | ON (6/8) |
| **Outer Volume Suppression (OVS)** | ON |
| **OVS Saturation Slab** | 150 mm (each side) |
| **CAIPIRINHA Shifts** | 2π/3 (1/3 FOV) |

**Supporting Table S4:** Implementation details of the numerical phantom. Other imaging parameters were set to match with the in-vivo experiments as in **Supporting Table S1.** For the reconstruction experiments, the in vivo trained models were used for both PG-DL methods with conventional and SIIM encoding, which also test their generalizability to a phantom with different image features than in vivo images. Thus, no additional trainings were performed. Rest of the reconstruction techniques were performed with the details given in **Supporting Table S3.** Peak signal-to-noise ratio (PSNR) and structural similarity index measure (SSIM) were calculated between single-band fully sampled reference images and all reconstruction techniques. Error images were calculated as the difference between reference images and the reconstructed images.


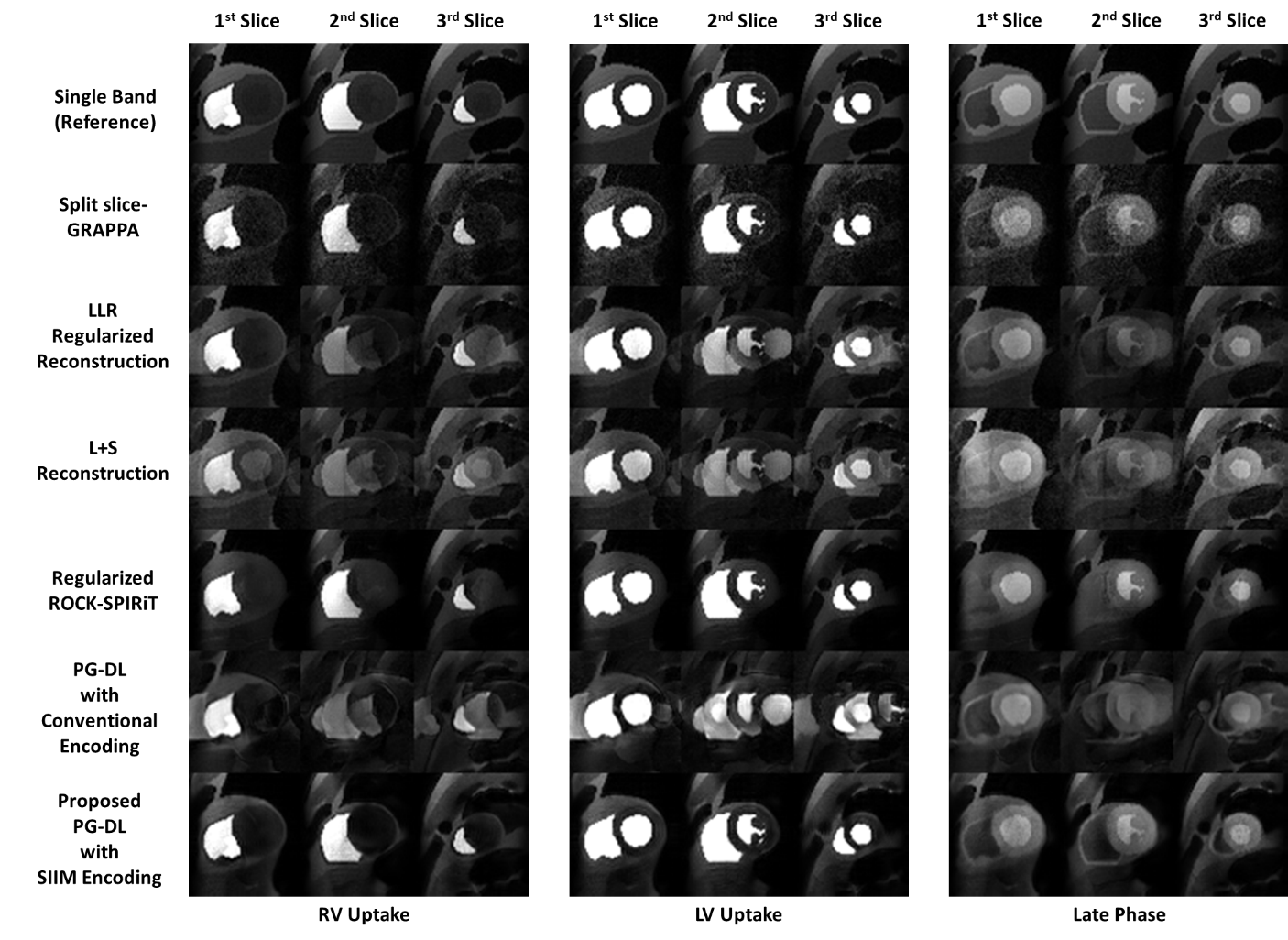


**Supporting Figure S5:** Representative numerical phantom results for myocardial perfusion CMR simulated with a 3-fold SMS and 4-fold in-plane acceleration along with outer volume suppression. Results across three different time frames are depicted along with single band reference images in the top row. The models trained on in-vivo datasets are used for PG-DL with conventional encoding and proposed SIIM encoding results without additional trainings. Compared to single-band fully sampled reference images (top row), proposed PG-DL with SIIM encoding operator shows the best visual image quality and improves upon all techniques. Split slice-GRAPPA does not show aliasing artifacts but suffers from noise amplification. Although LLR-regularized reconstruction, L+S reconstruction and PG-DL with conventional encoding reduce the noise, they all show aliasing artifacts. Furthermore, regularized ROCK-SPIRiT shows reduced noise, but residual artifacts are visible in right-ventricular at late phases.


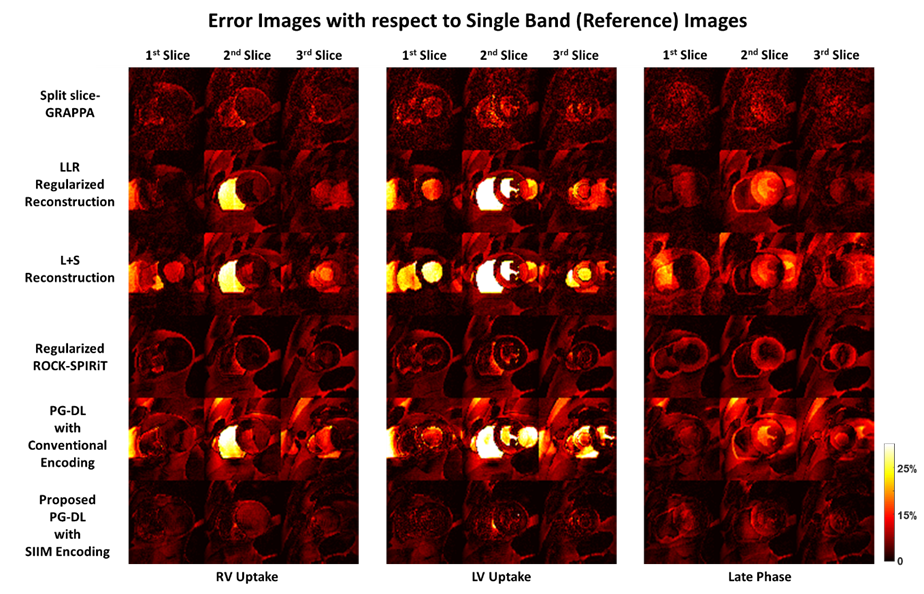


**Supporting Figure S6:** The error images between full-sampled reference images and all reconstruction methods for the numerical phantom experiments. The smallest difference is observed with the proposed technique where PG-DL with conventional encoding, L+S reconstruction and LLR-regularized reconstruction show substantial amount of residual artifacts. On the other hand, split slice-GRAPPA shows noise like difference without residual artifacts, whereas regularized ROCK-SPIRiT shows structural differences, especially around the myocardium periphery, indicating blurring in reconstruction.


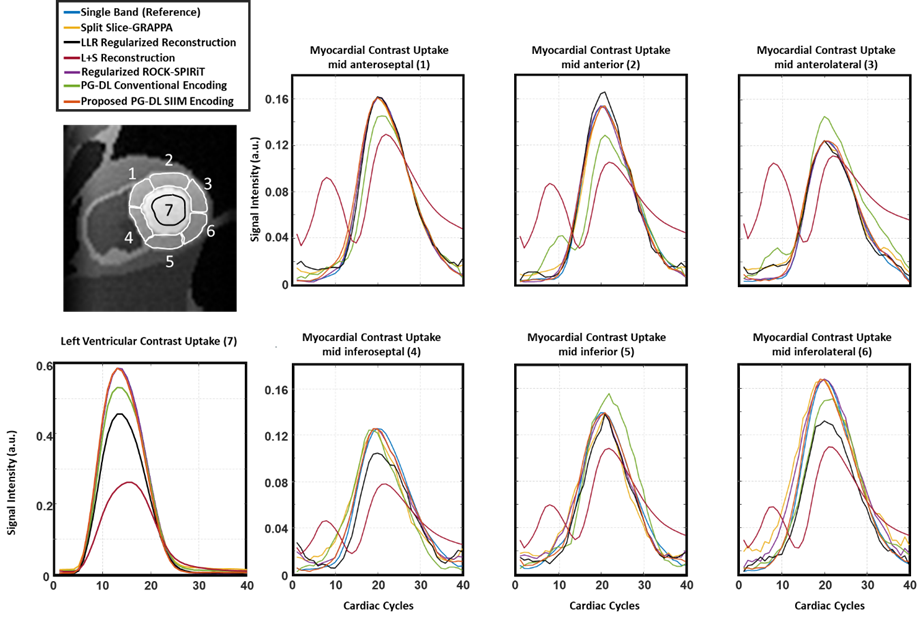


**Supporting Figure S7:** Signal intensity plots of left ventricular contrast uptake and 6 AHA segments of myocardial contrast uptake for all methods. A slice of interest for different sections of the myocardium (enumerated white regions) and the blood pool (enumerated black region) of the left ventricular is shown. Left ventricular uptake plot shows proposed method, split slice-GRAPPA and regularized ROCK-SPIRiT match well with the ground truth, whereas other methods fail to follow the uptake curve mainly due the residual artifacts shown in the Supporting Figures S1 and S2. Myocardial contrast uptakes of 6 AHA sections are plotted in the 2 by 3 grid next to the slice of interest where proposed method closely follows the ground truth. Similarly, in myocardial contrast uptake, LLR-regularized, L+S and PG-DL with conventional encoding reconstruction techniques fail and show major differences. On the other hand, split slice-GRAPPA shows similar uptakes compared to the ground truth, although it presents higher signal intensity levels especially in the early times frames mainly due to higher noise floor in the early time-frames in the mid anterolateral, mid inferolateral and mid inferior segments., ROCK-SPIRiT shows similar uptakes compared to the ground truth in mid anteroseptal and mid anterolateral segments, yet it presents significant misestimations in the rest of the segments.


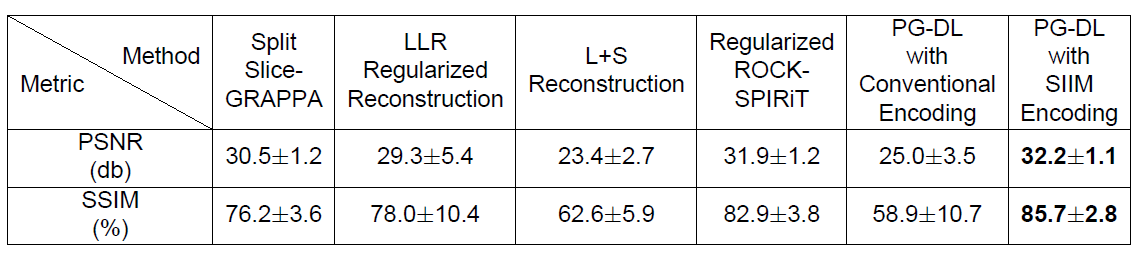


**Supporting Table S5:** Image quality assessment scores using PSNR and SSIM for all reconstruction methods using the numerical phantom. Proposed PG-DL with SIIM encoding shows the highest PSNR and SSIM values among all methods significantly improved upon clinical baseline split slice-GRAPPA with 1.7dB PSNR gain and 9.5% higher SSIM.

1. **Supporting Information for In Vivo Experiments**


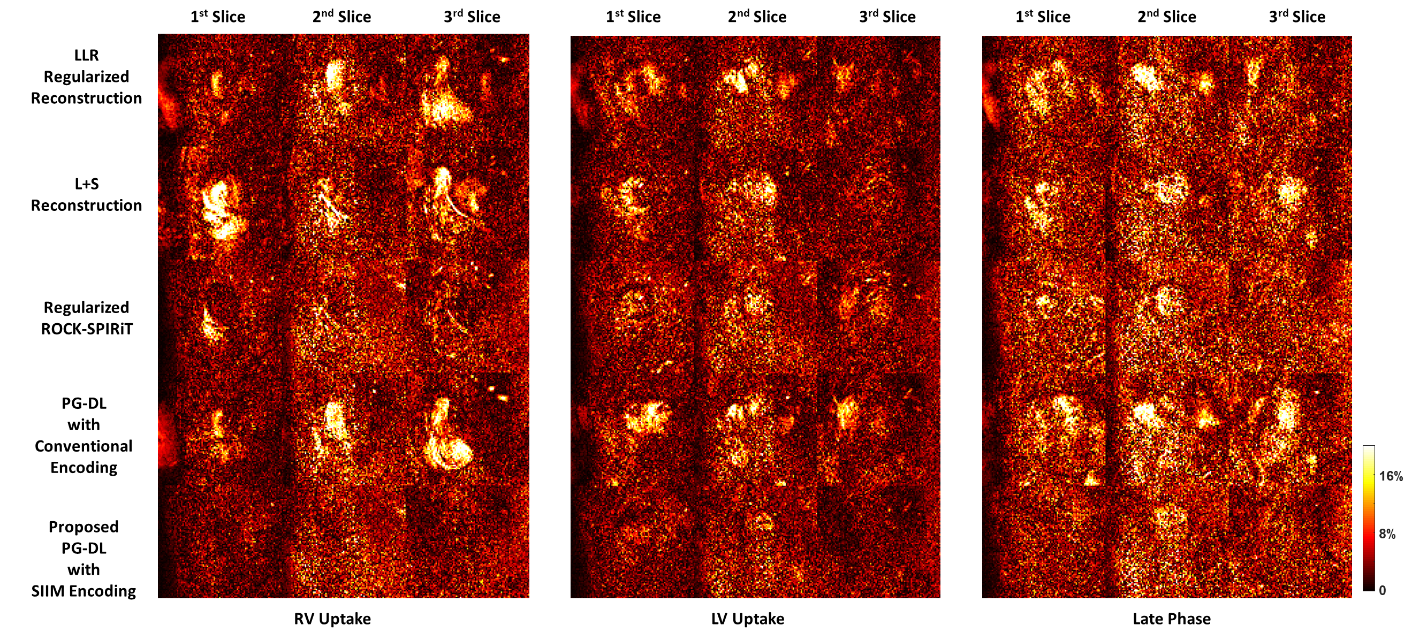


**Supporting Figure S8:** Difference images between linear baseline reconstruction split slice-GRAPPA and all the regularized reconstruction methods. Due to the lack of reference data in highly accelerated myocardial perfusion, error images were calculated with respect to slice-GRAPPA, where noise-like differences are expected with respect to this linear baseline calculation. LLR regularized reconstruction, L+S reconstruction, regularized ROCK-SPIRiT and PG-DL with conventional encoding all exhibit residual artifacts in the difference images, whereas in PG-DL with proposed SIIM encoding operator only noise is visible.


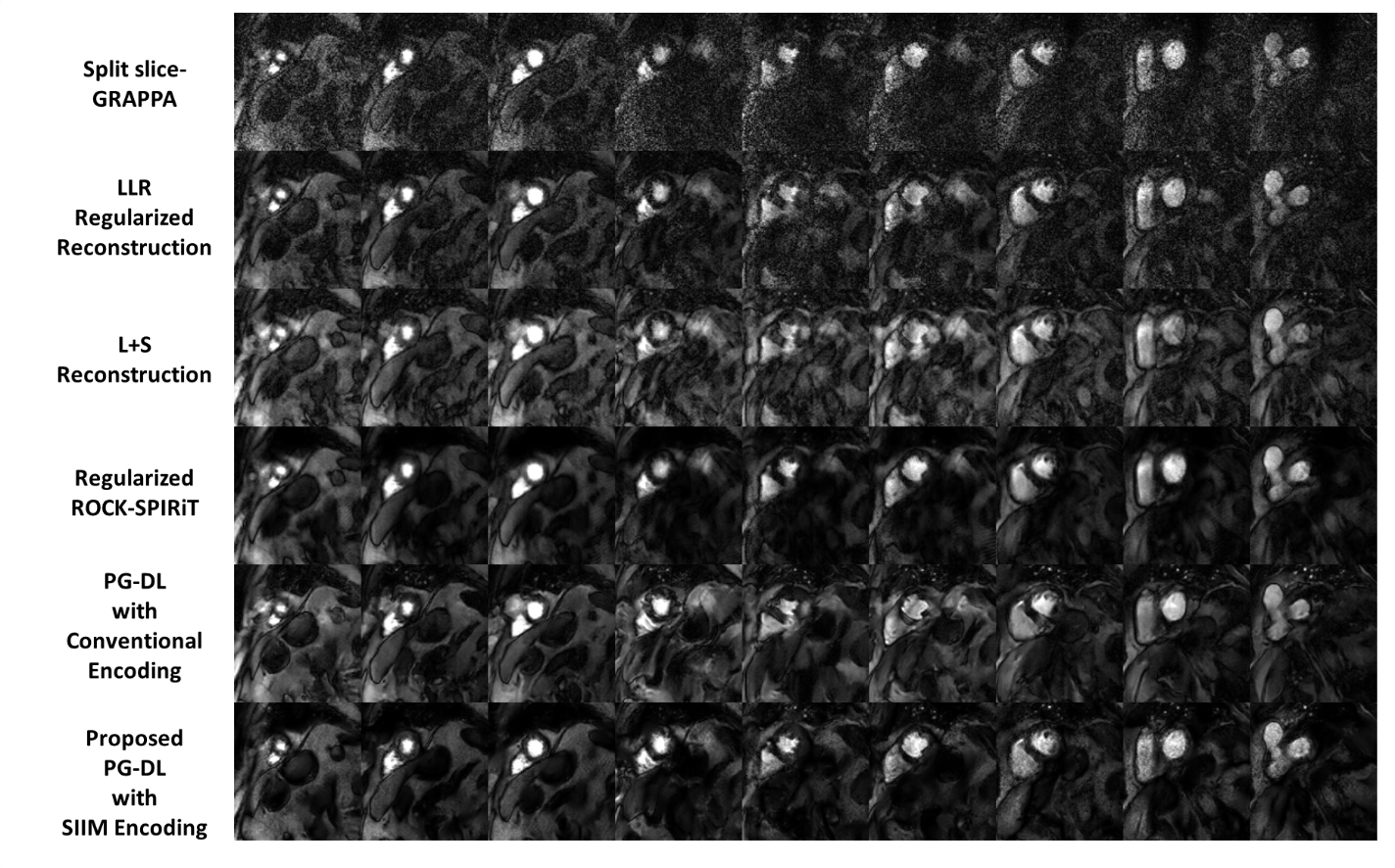


**Supporting Figure S9:** All 9 slices for a representative time-frame, covering the whole heart using 3 SMS groups of 9 slices from another subject shown in **Figure 3**.

*
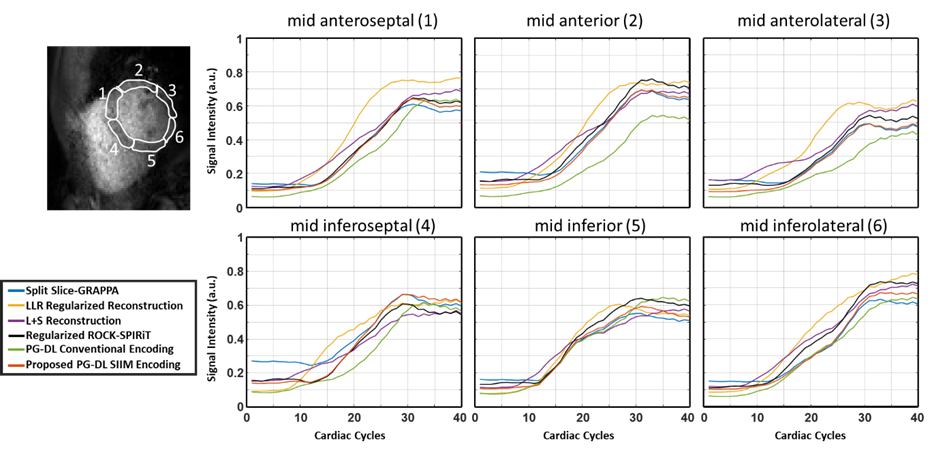
*

**Supporting Figure S10:** Low-pass filtered signal intensity curves of hand-drawn 6 AHA sectors in myocardium. Proposed PG-DL with SIIM encoding operator and split slice-GRAPPA are generally in line, with visible differences in early time frames where the noise level is higher for split slice-GRAPPA. Rest of the methods show visible misestimations compared to split slice-GRAPPA and the proposed method, albeit at different parts of the uptake curve.

**Supporting Video S1:** Movie of the perfusion images reconstructed with all techniques of a test subject (shown in Figure 2).

**Supporting Video S2:** Movie of the perfusion images reconstructed with all techniques of another test subject.

**References**

1. Fessler JA. Optimization Methods for Magnetic Resonance Image Reconstruction: Key Models and Optimization Algorithms. IEEE Signal Processing Magazine 2020;37(1):33-40.

2. Yaman B, Hosseini SAH, Moeller S, Ellermann J, Ugurbil K, Akcakaya M. Self-supervised learning of physics-guided reconstruction neural networks without fully sampled reference data. Magnetic Resonance in Medicine 2020;84(6):3172-3191.

3. Aggarwal HK, Mani MP, Jacob M. MoDL: Model-Based Deep Learning Architecture for Inverse Problems. IEEE Transactions on Medical Imaging 2019;38(2):394-405.

4. Weingärtner S, Moeller S, Akcakaya M. Feasibility of Ultra-high Simultaneous Multi-slice and In-plane Accelerations for Cardiac MRI Using Outer Volume Suppression and Leakage-Blocking Reconstruction. 2018. Annual Meeting of the International Society of Magnetic Resonance in Medicine.

5. Demirel OB, Yaman B, Dowdle L, Moeller S, Vizioli L, Yacoub E, Strupp J, Olman CA, Ugurbil K, Akcakaya M. Improved Simultaneous Multi-Slice Functional MRI Using Self-supervised Deep Learning. 55th Asilomar Conference on Signals, Systems, and Computers. IEEE, 2021; 890-894.

6. Demirel OB, Yaman B, Dowdle L, Moeller S, Vizioli L, Yacoub E, Strupp J, Olman CA, Ugurbil K, Akcakaya M. 20-fold Accelerated 7T fMRI Using Referenceless Self-Supervised Deep Learning Reconstruction. 43rd Annual International Conference of the IEEE Engineering in Medicine & Biology Society (EMBC). IEEE, 2021; 3765-3769

7. Demirel OB, Weingartner S, Moeller S, Akcakaya M. Improved simultaneous multislice cardiac MRI using readout concatenated k-space SPIRiT (ROCK-SPIRiT). Magnetic Resonance in Medicine 2021;85(6):3036-3048.

8. Yaman B, Hosseini SAH, Moeller S, Ellermann J, Ugurbil K, Akcakaya M. Multi-mask self-supervised learning for physics-guided neural networks in highly accelerated magnetic resonance imaging. NMR Biomed. 2022 Jul 5:e4798. doi: 10.1002/nbm.4798. Epub ahead of print. PMID: 35789133.

9. Timofte R, Agustsson E, Van Gool L, Yang MH, Zhang L, Lim B, Son S, Kim H, Nah S, Lee KM, Wang XT, Tian YP, Yu K, Zhang YL, Wu SX, Dong C, Lin L, Qiao Y, Loy CC, Bae W, Yoo J, Han Y, Ye JC, Choi JS, Kim M, Fan YC, Yu JH, Han W, Liu D, Yu HC, Wang ZY, Shi HH, Wang XC, Huang TS, Chen YJ, Zhang K, Zuo WM, Tang ZM, Luo LK, Li SH, Fu M, Cao L, Heng W, Bui G, Le T, Duan Y, Tao DC, Wang RX, Lin X, Pang JX, Xu JC, Zhao Y, Xu XY, Pan JS, Sun DQ, Zhang YJ, Song XB, Dai YC, Qin XY, Huynh XP, Guo TT, Mousavi HS, Vu TH, Monga V, Cruz C, Egiazarian K, Katkovnik V, Mehta R, Jain AK, Agarwalla A, Praveen CVS, Zhou RF, Wen HD, Zhu C, Xia ZQ, Wang ZT, Guo Q, IEEE. NTIRE 2017 Challenge on Single Image Super-Resolution: Methods and Results. IEEE Computer Society Conference on Computer Vision and Pattern Recognition Workshops; 2017 Jul 21-26; Honolulu, HI. p 1110-1121.

10. Koolstra K, van Gemert J, Bornert P, Webb A, Remis R. Accelerating compressed sensing in parallel imaging reconstructions using an efficient circulant preconditioner for cartesian trajectories. Magnetic Resonance in Medicine 2019;81(1):670-685.
